# Supplementary material for: Machine learning-guided reconstruction of cytoskeleton network from live-cell AFM images
Source: iScience. 2024 Sep 10;27(10):110907. doi: 10.1016/j.isci.2024.110907 (PMC11465173; doi:10.1016/j.isci.2024.110907)
Supplement: Document S1. Figures S1–S3 [file mmc1.pdf]

iScience, Volume *27*

Supplemental information

Machine learning-guided reconstruction of cytoskeleton network from live-cell AFM images

Hanqiu Ju, Henrik Skibbe, Masaya Fukui, Shige H. Yoshimura, and Honda Naoki

Supplemental information

Supplemental Figures:

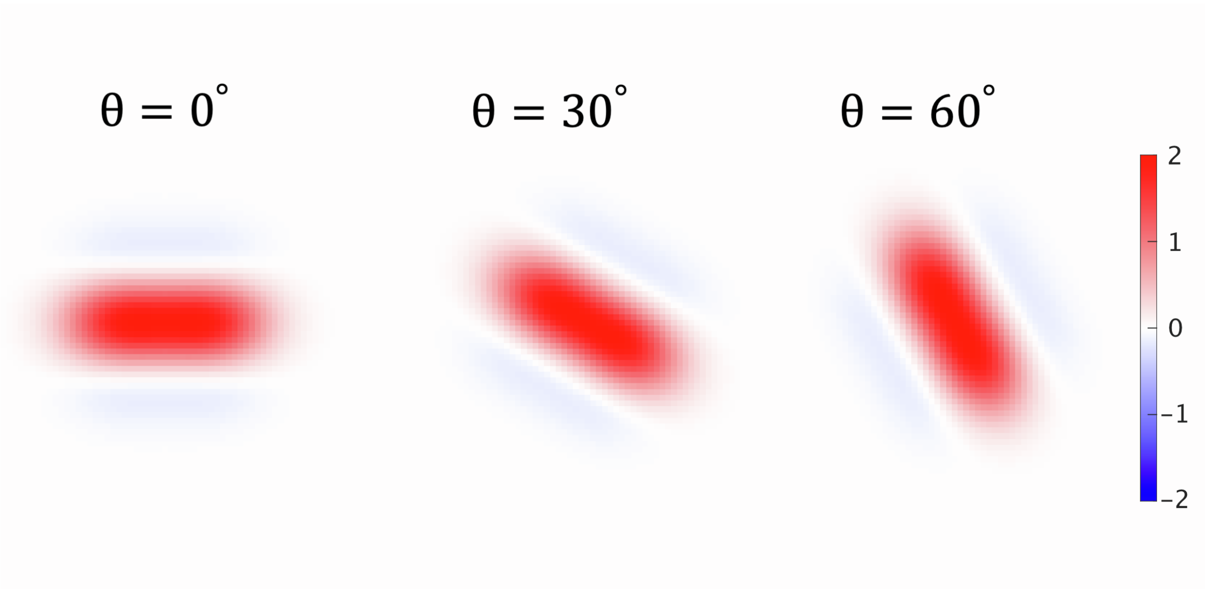

Figure S1. Shape of the linear mapping kernel, Related to equation (12).
Shape of the real part of the kernel described in Eq. 12 with $\theta = 0^o, 30^o$ and 60°. The real part of Eq. 12 is the tubular structure detection, which can detect tubular structure based on the θ orientation.

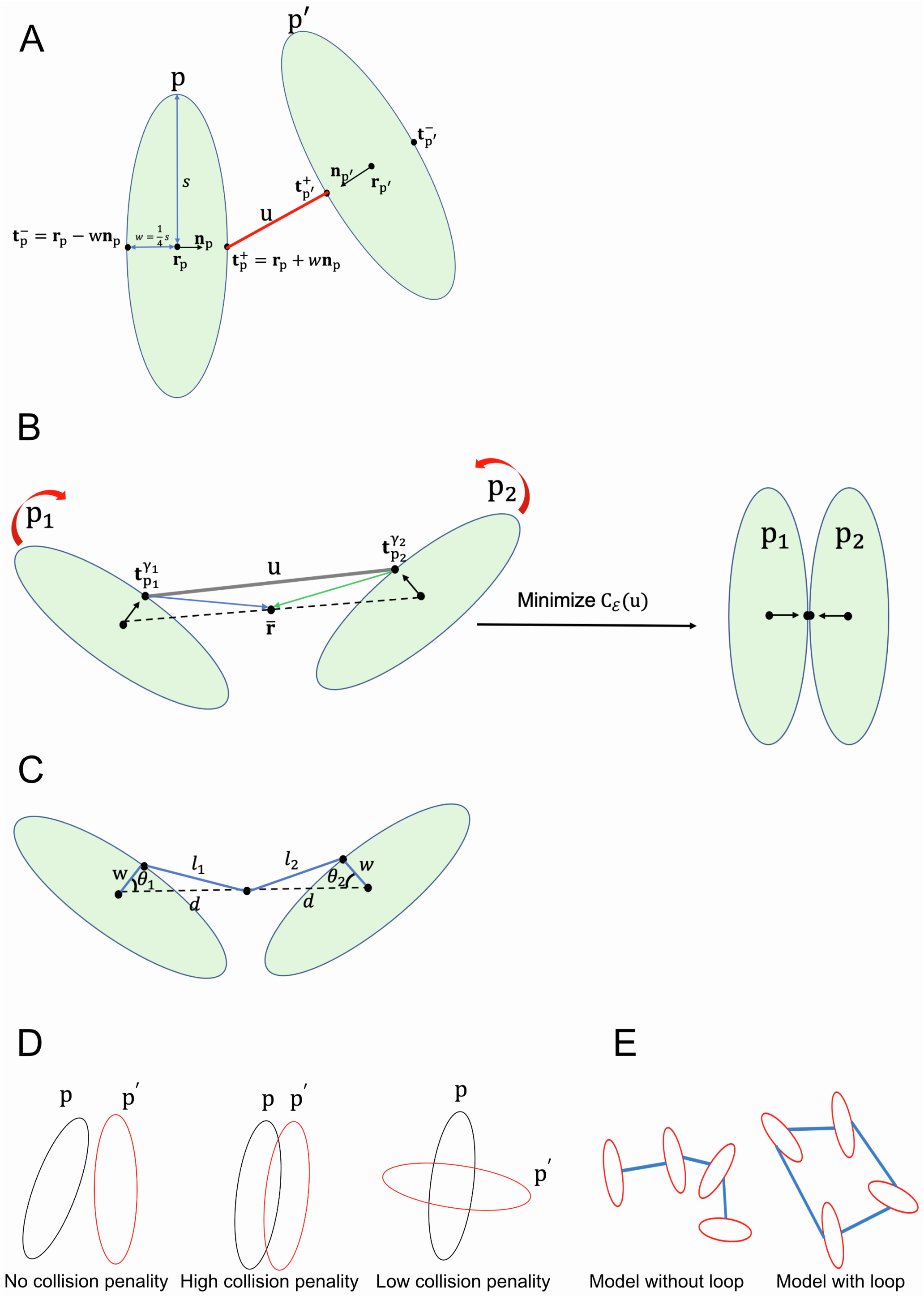

Figure S2. Graphical descriptions of the internal energies, Related to equations (16-19).

(A) Definitions of the attributes of ellipse-shaped particles. A particle p is defined by its position $\boldsymbol{r}_p$ and orientation $\boldsymbol{n}_p$, where scale (s) and thickness (w) are constants shared among all the particles. The edge (u) is depicted by the red line and defined as follows, $u = (\boldsymbol{t}_p^+, \boldsymbol{t}_{p'}^+)$, where $\boldsymbol{t}_p^+$ and $\boldsymbol{t}_p^-$ are coordinates of the particle connecting sites. **(B)** Elastic energy of the F-actin represented by Eq. 16. The pulling force is generated so as to minimize the squared sum of lengths of the blue and green lines $(l_1^2 + l_2^2)$, which are respectively segments from the midpoint between the central points of p_1 and p_2 $(\bar{\boldsymbol{r}})$ to the particles' connecting sites $(\boldsymbol{t}_{p_1}^{\gamma_1}$ and $\boldsymbol{t}_{p_2}^{\gamma_2})$. The red curve arrows represent the additional bending energy term described in Eq. 16 to strengthen the elastic penalty by penalizing the differences between

the orientations of p_1 and p_2. **(C)** Geometric interpretation of the bending energy of F-actin represented by Eq. 16. **(D)** Collision energy between two particles. The left, middle, and right show no collision energy between the non-overlapping particles, high collision energy between the overlapping particles, and low collision energy between the orthogonally overlapping particles, respectively. **(E)** Loop energy. There is no loop energy without a closed loop (left). The loop energy is infinite with a closed loop, which means that a model with a closed loop should be discarded (right).

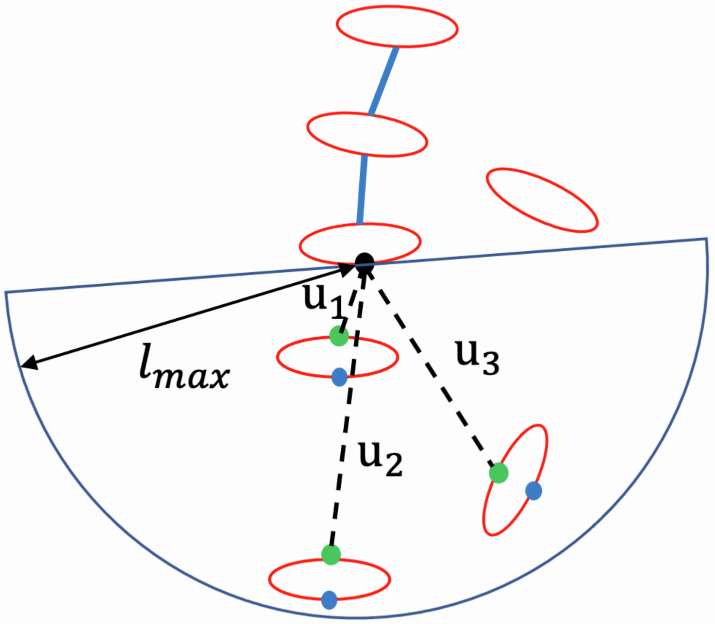

Figure S3. Creation of candidate edges in the connection/reconnection proposal, Related to equation (30).

Connection or reconnection sites were randomly selected (black points). Candidate edges $\varepsilon^{\text{cand}}$ were searched for within a semicircle area with a radius of l_{max}, which was generated from the end particle toward its orientation. The free connecting sites facing the selected connecting site (green points) are candidates, whereas those facing the opposite direction (blue points) are ignored. The candidate edge with minimal elastic energy was selected as the new edge for MCMC sampling. In this case, u_1 is selected.
